# Correlation of ERCC5 polymorphisms and linkage disequilibrium associated with overall survival and clinical outcome to chemotherapy in breast cancer

**DOI:** 10.3389/fonc.2022.1091514

**Published:** 2023-01-04

**Authors:** Iqra Khan, Nosheen Masood, Azra Yasmin

**Affiliations:** Department of Biotechnology, Fatima Jinnah Women University, Rawalpindi, Pakistan

**Keywords:** breast cancer, ERCC5 gene, survival analyses, linkage disequilibrium, polymorphism

## Abstract

**Purpose:**

ERCC5 is a DNA endonuclease and nucleotide excision repair gene; its mutations lead to a lack of activity by this enzyme, causing oxidative DNA damage. This study aimed to assess the role of four selected single nucleotide polymorphisms (SNPs) in ERCC5 and their linkage disequilibrium associated with survival analysis and clinical outcomes in breast cancer.

**Patients and methods:**

Four SNPs (rs751402, rs17655, rs2094258, and rs873601) of the ERCC5 gene were analyzed using the PCR-RFLP technique, followed by sequencing in 430 breast cancer (BC) cases and 430 cancer-free individuals. Statistical analysis was performed using MedCalc 17 and SPSS version 24, while bioinformatic analysis of linkage disequilibrium was performed using Haploview software 4.2.

**Results:**

Multivariate analysis showed that the rs751402 and rs2094258 polymorphisms were significantly associated with an elevated risk of BC (P < 0.001), while the other two SNPs, rs17655 and rs873601, did not show any association (P > 0.001). Survival analysis revealed that rs751402 and rs2094258 had longer overall survival periods (P <0.001) than rs17655 and rs873601. Moreover, rs751402 and rs2094258 also had significantly longer overall survival (log-rank test, P < 0.005) for all three survival functions (positive family history, ER+PR status, and use of contraceptives), while rs17655 and rs873601 did not show any significant association. Only rs873601 showed a strong negative correlation with all the chemotherapeutic groups.

**Conclusion:**

The current results suggest that variations in ERCC5 may contribute to BC development and that their genetic anomalies may be associated with cancer risk and may be used as a biomarker of clinical outcome.

## Introduction

Breast cancer (BC) is one of the most common malignancies and the primary cause of death among females worldwide (1). One in nine women in Pakistan faces this brutal disease (2). The mechanisms underlying breast carcinogenesis have not yet been fully explored and need to be completely understood. Various polymorphisms of genes involved in DNA damage responses play a significant role in cancer development and proliferation. Genes associated with DNA repair pathways are considered candidate genes for cancer susceptibility because reduced repair efficiency may induce carcinogenesis (3). One of the DNA repair pathways is the nucleotide excision repair (NER) pathway, which is significantly associated with cancer risk. Maintaining genomic stability and preventing the propagation of errors in the genome requires efficient DNA repair, and the NER pathway helps in the repair of bulky lesions such as thymine dimers generated by ultraviolet radiation (4). ERCC5 is a vital constituent of the NER mechanism and is called xeroderma pigmentosum group G (XPG). It encodes an endonuclease enzyme, which makes a structure-specific 3’- incision at damaged DNA sites. It can also act non-enzymatically by participating in a 5’ incision with the help of the ERCC1/XPF heterodimer (5). ERCC5 is expressed in different tissues and cell lines, and its deficiency leads to genomic instability, DNA repair faults, and non-functioning gene transcription modulation and thus plays a role in DNA damage and higher breast cancer susceptibility, and regulation of DNA repair is a vital feature in various steps of carcinogenesis. Single nucleotide polymorphisms in ERCC5 may change its activity or expression, affecting DNA repair function, resulting in the alteration of cancer treatment effects, as treatment outcomes depend on the genetic variant of the gene present (6, 7).

Many studies have depicted that XPG polymorphisms are linked with various cancers like gastric, lung, breast, and colorectal (8–11). However, to our knowledge, only a limited number of studies have been conducted on the association analysis of these particular polymorphisms of ERCC5 (rs751402, rs17655, rs2094258, and rs873601) in BC patients and their response to chemotherapy. To investigate the possible influence of ERCC5 on BC, a case-control study was designed to evaluate the active involvement of these selected polymorphisms. Our study highlights the correlation of ERCC5 polymorphisms with various clinicopathological factors, overall survival rates with different survival functions, linkage disequilibrium analysis, and therapeutic outcomes of different chemotherapeutic drugs among breast cancer patients. Linkage disequilibrium analysis was conducted to explore the combined effects of these ERCC5 germline variants on breast carcinogenesis. It is expected that the data generated in the present study will help health practitioners make treatment decisions or provide the best advice based on an assessment of risk.

## Materials and methods

### Subjects and ethical considerations

The study was approved by the ethical committees of the Institute of Nuclear Medicine, Oncology, and Radiotherapy (INOR) Hospital, Abbottabad, Pakistan, and Fatima Jinnah Women University, Rawalpindi, Pakistan. The sample size was evaluated using a sample size calculator provided by the World Health Organization and validated manually by. Blood samples and demographic details were collected from 430 histologically confirmed breast cancer patients (mean age 47.32 ± 11.7) and healthy controls (mean age 46.3 ± 14.03, P = 0.005), with patients’ consent signed by them to participate in the study (2019–2022). A questionnaire was designed for the collection of clinicopathological details of patients.

### Single nucleotide polymorphism selection

Four potential SNPs (rs751402, rs17655, rs2094258, and rs873601) were selected from the National Center for Biotechnology Information SNP data base (http://www.ncbi.nlm.nih.gov/) and SNPinfo (http://snpinfo.niehs.nih.gov/) combined with previously described studies on the characteristics of the East Asian population in HapMap with minor allele frequency (MAF >5%). SNP rs17655 is a non-synonymous SNP (nsSNP) present in exon 15, while the remaining three SNPs are present in the regulatory region of ERCC5 (i.e., the 3′ untranslated region (UTR), the 5′ UTR promoter region, and the 5′ near gene). rs2094258 in the 5′ near gene was predicted to affect transcription factor binding site activity, rs751402 was present in the 5’ UTR promoter region of the gene, and rs873601 in the 3’UTR may have an influence on the splicing and miRNA binding sites.

### DNA extraction and polymorphism screening

Blood samples were collected in EDTA vacutainers and stored at −20°C until further use. Genomic DNA was isolated from blood samples by the standard phenol- chloroform method (5) and stored in a refrigerator at 4°C until further analysis. Qualitative analysis of DNA was performed using conventional electrophoresis on a 1% agarose gel and a spectrophotometer. Genotyping of the ERCC5 germline variants rs751402, rs17655, rs2094258, and rs873601 was performed by polymerase chain reaction-restriction fragment length polymorphism (PCR-RFLP), following a method modified by Guo et al. (12). Primers were obtained from the published literature and are listed in **Supplementary Table S1** along with their respective references.

### Statistical and survival analysis

Clinicopathological details, demographic characteristics, and ERCC5 variants between BC patients and healthy controls were analyzed using Pearson’s chi- square test (χ^2^) and Fisher’s exact test. Conditional logistic regression was applied to find the associations between ERCC5 SNPs, clinicopathological details, and breast cancer risk by computing 95% confidence intervals (95% CIs) and odds ratios (ORs). Frequency distribution analysis was performed according to Hardy–Weinberg equilibrium (HWE) statistics. Patient follow- up was performed every 6 months and the homozygous wild variant was taken as a reference in all four ERCC5 SNPs. The overall survival (OS), survival distributions, and OS with three survival functions were estimated using Kaplan–Meier and log- rank tests. The survival distributions among the different classes of chemotherapy drugs were also assessed. Patients were classified based on the chemotherapeutic drugs administered. Taxanes, cytotoxic agents, and a combination of chemotherapeutic drugs were administered to all patients treated with chemotherapy. The frequency of chemotherapeutic drugs in breast cancer with both SNPs was analyzed by the chi- squared test. The correlation between SNPs and chemotherapeutic drugs was also assessed. Linkage disequilibrium analysis was performed using Haploview software 4.2. Significance level was set at P <0.05. All statistical analyses were performed using IBM SPSS version 24 and MedCalc 17.

## Results

### Subject characteristics

The current study aimed to assess the genetic variations in the DNA excision repair protein ERCC-5 of the nucleotide excision repair pathway in 430 BC patients and 430 healthy controls. The demographic details and genotype frequencies of ERCC5 in patients with BC and healthy controls are shown in **Table 1**. The demographic parameters studied included family history, age, cancer staging, chemotherapeutic drug type, menopausal status, BMI, treatment type, marital status, and age at menarche. Most BC patients had stage III (39.4%) cancer, while only 16.2% had stage I cancer. Approximately 41.2% and 54.9% of the patients were treated with radiotherapy and chemotherapy, respectively. The data showed that the mean BMI for cases was 27.96 ± 5.64, showing obesity as a risk factor for BC (OR = 1.07, 95% CI = 2.62–3.65). Age, age at menopause, and menarche were evaluated as risk factors for BC (P <0.001). Significant differences were observed in marital status (OR = 0.4, 95% CI = 0.14–0.11, P <0.001), family history (OR = 0.28, 95% CI = 0.1–0.4, P <0.001), and menopausal status (OR = 1.61, 95% CI = 1.27–2.1, P <0.001) (**Table 1** **)**.

**Table 1:** Frequency distribution of demographic factors, chemotherapeutic drugs, and ERCC5 germline variants in BC patients and controls.

Characteristics	Cases		Controls	
	Frequency	Percent	Frequency	Percent
Age groups				
**15–30**	30	3.5	97	11.3
**31–45**	172	20	314	36.5
**46–60**	177	20.6	328	38.1
**61–85**	51	5.9	121	14.1
Marital status				
**Unmarried**	82	95.3	4	4.7
**Married**	348	45	426	55
Family History				
**No**	381	56.3	296	43.7
**Yes**	49	26.8	134	72.2
Menopausal status				
**Premenopausal**	270	55.1	220	44.9
**Postmenopausal**	160	43.2	210	56.8
Chemotherapeutic drugs				
**Cytotoxic**	67	77.8		
**Taxanes + Cytotoxic**	134	15.6		
**Cytotoxic + others**	20	2.3		
**Taxanes + others**	13	1.5		
**Cytotoxic + Taxanes + others**	2	0.2		
rs17655				
**CC**	343	79.8	407	94.7
**CG**	80	18.6	23	5.3
**GG**	7	1.6		
rs751402				
**GG**	66	15.3	323	75.1
**AG**	101	23.5	62	14.4
**AA**	263	61.2	45	10.5
rs2094258				
**GG**	200	46.5	269	62.6
**AG**	60	14	61	14.2
**AA**	107	39.5	100	23.3
rs873601				
**AA**	300	69.8	280	65.1
**AG**	80	18.6	70	16.3
**GG**	50	11.6	80	18.6

### Association of ERCC5 germline variants and clinicopathological parameters

To associate the genotype frequency of the assessed SNPs with clinicopathological factors, we applied logistic regression and χ2 tests. In this analysis, clinicopathological factors such as family history, marital status, ER status, PR status, and menopausal status were considered independent factors, and the genotype of all evaluated SNPs was considered a dependent variable, as illustrated in **Table 2**. The distribution frequency of the homozygous variant type and heterozygous variant type of ERCC5 rs751402 was only associated with patients who used contraceptives rather than the homozygous wild type (OR = 2.8; 95% CI = 1.6–4.9; P = 0.0003). The heterozygous and homozygous variant types of ERCC5 rs17655 were associated with patients who had a positive family history of cancer (OR = 2.1, 95% CI = 0.9–5.04; P = 0.05). There were no statistically significant correlations between the genotype distributions of both SNPs and menopausal, ER/PR, or marital status (**Table 2**). However, the analysis revealed a strong negative correlation between rs873601 and positive ER/PR status (OR = 3.45, 95% CI = 2.57–4.61; P = −0.04), rs873601 versus contraceptive use (OR = 1.69; 95% CI = 1.07–2.67; P = −0.09), and rs873601 versus menopausal status (OR = 3.35; 95% CI = 2.40–4.66; P = −0.005). No significant correlation was observed between rs873601 and family history (OR = 0.24; 95% CI = 0.15–0.37; P = 0.24), but married women showed a positive correlation (OR = 5.26; 95% CI = 3.93–7.04; P = 0.04) (**Table 2** **)**. The fourth selected polymorphism rs2094258 showed a strong positive correlation with ER/PR status (OR = 1.23; 95% CI = 0.93–1.62; P = 0.01) and use of contraceptives (OR = 1.07, 95% CI = 0.69–1.68; P = 0.01), whereas a strong negative correlation was observed with marital status (OR = 1.03; 95% CI = 0.79–1.35; P = −0.007), family history (OR = 1.19; 95% CI = 0.82–1.73; P = −0.004), and menopausal status (OR = 1.15, 95% CI = 0.84–1.57; P = −003) (**Table 2**).

**Table 2:** Correlations between clinicopathological parameters and ERCC5 germline variants in patients with breast cancer (n = 430).

SNPs vs Parameters	Homozygous wild type No (%)	Variants types (homozygous, heterozygous) No (%)	P	OR (95% CI)	Z test
ERCC5 rs751402 vs					
Menopausal status	37 (56.1)	183 (50.3)	0.38	1.2 (0.7–2.1)	0.8
Premenopausal					
Postmenopausal	29 (43.9)	181 (49.7)	0.3	0.79 (0.46–1.34)	0.86
Family History	18 (27.3)	114 (31.3)	0.5	0.8 (0.4–1.4)	0.65
Positive ER/PR status	45 (68.2)	282 (77.5)	0.1	0.6 (0.3–1.1)	1.6
Contraceptive use	25 (36.4)	63 (17.1)	0.0003	2.8 (1.6–4.9)	3.6
Married	64 (97)	361 (99.4)	0.1	0.2 (0.04–1.6)	1.4
ERCC5 rs17655 vs					
Menopausal status					
Premenopausal	206 (50.6)	14 (60.9)	0.3	0.6 (0.2–1)	0.95
Postmenopausal	201 (49.4)	9 (39.1)	0.4	0.65 (0.27–1.55)	0.9
Family History	121 (29.7)	11 (47.8)	0.07	2.1 (0.9–5.04)	1.79
Positive ER/PR status	311 (76.4)	16 (69.6)	0.4	0.7 (0.2–1.7)	0.7
Contraceptive use	84 (20.7)	2 (8.7)	0.1	0.3 (0.08–1.5)	1.4
Married	404 (99)	22 (100)	0.6	0.5 (0.02–9.5)	0.4
ERCC5 rs2094258 vs					
Menopausal status					
Premenopausal	102 (51)	118 (51.3)	0.9	0.9 (0.6–1.4)	0.06
Postmenopausal	98 (49)	112 (48.7)	0.9	1.15 (0.84–1.57)	0.9
Family History	61 (30.5)	71 (30.9)	0.9	1.19 (0.82–1.73)	0.94
Positive ER/PR status	153 (76.5)	174 (75.7)	0.8	1.23 (0.93–1.62)	1.47
Contraceptive use	42 (20.61)	45 (19.7)	0.7	1.07 (0.69–1.68)	0.33
Married	198 (1)	228 (99.1)	0.8	1.03 (0.79–1.35)	0.2
ERCC5 rs873601 vs					
Menopausal status					
Premenopausal	153 (51)	67 (51.5)	0.6	0.9 (0.6–1.3)	0.4
Postmenopausal	157 (49)	63 (48.5)	0.8	3.35 (2.4–4.66)	7.14
Family History	102 (34)	30 (23.1)	0.02	0.24 (0.15–0.37)	6.44
Positive ER/PR status	224 (74.7)	103 (79.2)	0.2	3.45 (2.57–4.61)	8.33
Contraceptive use	53 (17.7)	33 (25.6)	0.03	1.69 (1.07–2.67)	2.25
Married	297 (99.3)	128 (98.5)	0.47	5.26 (3.93–7.04)	11.2

### Linkage disequilibrium analysis

**Figure 1:**
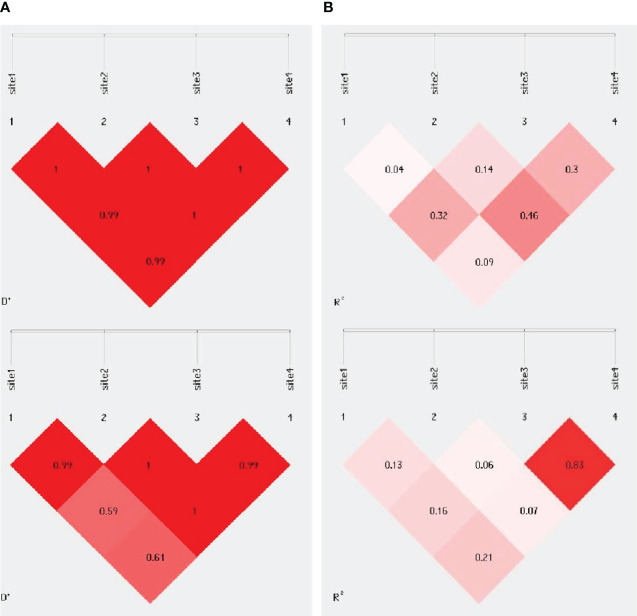
Pairwise linkage disequilibrium plot for evaluated ERCC5 polymorphism in **(A)** controls **(B)** BC cases. Site 1 is for rs751402, Site 2 is for rs17655, Site 3 for rs20942584 for rs873601. The darker area indicates higher D’ & r^2^ value.

The analysis of linkage disequilibrium of the evaluated polymorphism of the ERCC5 gene was calculated using Haploview software, as shown in **Figure 1**. LD values are displayed as r2 and D values. Site 1 represents rs751402, site 2 represents rs17655, site 3 represents rs2094258 and site 4 represents rs873601. Sites 3 and 4 (rs2094258 and rs873601, respectively) exhibited a stronger association with LD among cancer patients than among healthy controls.

### ERCC5 variants, survival distributions, and overall survival of breast cancer patients

Kaplan–Meier survival analysis and the log- rank test were used to determine the association between ERCC5 germline variants and overall survival (OS) **(** **Table S2** **)**. The rs751402 and rs2094258 polymorphism showed significant association (Log-rank test, P <0.001; Mean GG = 12, AA/AG = 22.3; 95% CI (GG) = 8.7–15.2, AA/AG = 20.8–23.8) (Log-rank test, P = 0.005; Median GG = 27 months, AG/AA = 21 months, 95% CI GG = 25.3–28.3, AA/AG = 16.4–25.5) (**Figure S1**) **(** **Table S2** **),** respectively, while the other two SNPs; rs17655 and rs873601, did not show any association with OS (Log-rank test, P = 0.3; Median CG + GG = 25; 95% CI = 20.3–29.6) (Log-rank test, P = 0.86; Median AA = 25 months, AG/GG = 26 months, 95% CI AA = 21.4–28.8, AA/AG = 16.4–35.5), respectively (**Figure S1**). Furthermore, we studied three survival functions: positive family history, ER/PR status, and contraceptive use, with all four evaluated SNP variants. It was found that rs751402 and rs2094258 had significantly longer OS for all three patient survival functions (log-rank test, P < 0.005). The estimated median for BC patients with a positive family history who had homozygous wild type rs751402 was 7 months (95% CI = 0.00–15.4), and for those with homozygous variant type and heterozygous variant type, it was 25 months (95% CI = 21.5–28.4) **(** **Figure S1** **),** and the estimated median having homozygous wild type with positive ER/PR status was 11 months (95% CI = 4.2–17.7), and for homozygous variant type and heterozygous variant type was 26 months (95% CI = 24.5–27.4) **(** **Figure S1** **)**. For patients with rs751402 who had used contraceptives, the estimated median was 13 months (95% CI = 9.3–16.6) for homozygous wild type, while for homozygous variant type and heterozygous variant type it was 26 months (95% CI = 24.2–27.75) **(** **Figure S1** **)**. No association was found for rs17655 and rs873601 with all three survival functions (log-rank test, P >0.5). For the patients with wild type for all three survival functions (positive family history, ER + PR status, and use of contraceptives) of rs17655, the median was 26 months (95% CI=19.2-32.7), 26 months (95% CI=21.5-30.4), and 25 months (95% CI=16.2-33.7), respectively, and for homozygous variant type and heterozygous variant type, the median was 21 months (95% CI = 15.1–26.8), 25 months (95% CI = 19.8–30.1), and 18 months (95% CI = 3.59–32.4), respectively **(** **Figure S1** **)**. Similarly, for the patients with homozygous variant type and heterozygous variant type of rs873601, the median for all three survival functions (positive family history, ER + PR status, used contraceptives) was 26 months (95% CI = 0.00–59.3), 27 months (95% CI = 15.9-38), and 18 months (95% CI = 6.3–29.6), respectively, and for the wild type, the median was 25 months (95% CI = 21.1–28.8), 25 months (95% CI = 21.5–28.4), and 25 months (95% CI = 17.8–32.1) **(** **Figure S1** **),** respectively. For taking into consideration survival functions, evaluation for rs2094258 (positive family history, ER + PR status, used contraceptives), the median for homozygous and heterozygous variant types was 21 months (95% CI = 15.2–26.7), 24 months (18.3–29.6), and 18 months (95% CI = 11.43–24.5), respectively, while for the wild type was 27 months (95% CI = 24.7–29.2), 27 months (95% CI = 25.2–28.7), and 27 months (95% CI = 16.4–24.5), respectively **(** **Figure S1** **)**.

### Survival distributions for different chemotherapeutic drugs classes

**Figure 2:**
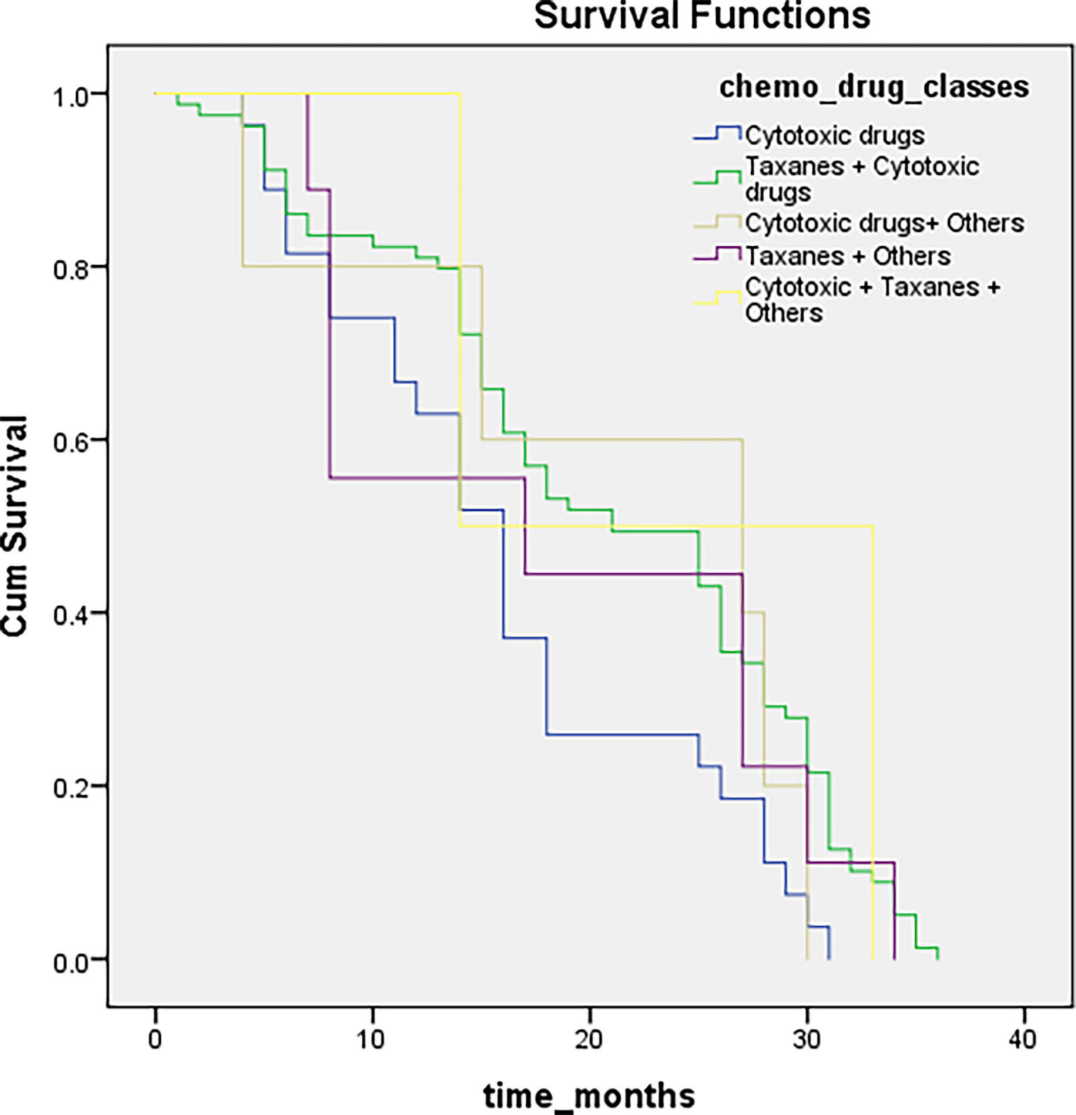
Illustrating survival distributions for different chemotherapeutic drugs classes among breast cancer patients.

Chemotherapeutic drug- related data were available for only 236 patients, possibly because they were not taking those drugs or had missing records from the files. We categorized all chemotherapeutic drugs given in different classes: cytotoxic drugs, taxanes, others, cytotoxic and taxanes, and all three were given together in combination. Patients were followed up every six months to inquire about their health condition, monitor the effectiveness of drugs, and for survival analysis. Genetic analysis was conducted to evaluate the association of SNPs with the response to a particular chemotherapeutic drug type. The outcomes are summarized in **Table 3**. We were unable to find any association between the respective chemotherapeutic drugs and rs17655 (P >0.001), whereas rs751402 showed a significant association (P <0.001). The overall survival for all the drugs administered was not statistically significant (log-rank test, P = 0.09). Survival differences for different drugs were compared through Breslow, Tarone–Ware, and the Log-rank test, which showed insignificant results for all drugs given (Log-rank test, P = 0.09; median = 18; 95% CI = 14.9–21.08) (**Figure 2**).

**Table 3:** Genotyping frequency and correlation of ERCC5 germline variants with chemotherapeutic drugs in breast cancer patients.

SNPs vs drug type	Homozygous wild typeNo (%)	Variants types (homozygous, heterozygous) No (%)	Chi-Square (χ^2^)	Pearson Correlation
**ERCC5 rs751402 vs**			P <0.001	0.2
Cytotoxic drugs	33 (53.3)	35 (19.9)		
Taxanes + Cytotoxic	19 (31.7)	115 (65.3)		
Cytotoxic + Others	9 (15)	11 (6.3)		
Taxanes + Others	0 (0)	13 (7.4)		
Cytotoxic + Taxanes + Others	0 (0)	2 (1.1)		
**ERCC5 rs17655 vs**			P >0.001	0.4
Cytotoxic drugs	63 (31.7)	4 (28.4)		
Taxanes + Cytotoxic	101 (50.8)	33 (89.2)		
Cytotoxic + Others	20 (10.1)	0 (0)		
Taxanes + Others	13 (6.5)	0 (0)		
Cytotoxic + Taxanes + Others	2 (1)	0 (0)		
**ERCC5 rs2094258 vs**			P = 0.1	P = 0.1
Cytotoxic drugs	39 (36.1)	28 (21.9)		
Taxanes + Cytotoxic	55 (50.9)	79 (61.7)		
Cytotoxic + Others	9 (8.3)	11 (8.6)		
Taxanes + Others	5 (4.6)	8 (6.3)		
Cytotoxic + Taxanes + Others	0 (0)	2 (1.6)		
**ERCC5 rs873601 vs**			P >0.001	−0.26
Cytotoxic drugs	32 (19.5)	35 (48.6)		
Taxanes + Cytotoxic	107 (65.2)	27 (37.5)		
Cytotoxic + Others	11 (6.7)	9 (12.5)		
Taxanes + Others	12 (7.3)	1 (1.4)		
Cytotoxic + Taxanes + Others	2 (1.2)	0 (0)		

## Discussion

The present study was designed to associate single nucleotide polymorphisms of ERCC5 (rs751402, rs2094258, rs17655, and rs873601) with breast cancer and associated risk factors. A significantly higher rate of variants at rs751402 and 2094258 was observed in breast cancer patients than in non-cancerous individuals, while the other two evaluated SNPs did not show any association. Only rs17655 was present in the exonic region, whereas the remaining three were present in the regulatory region of ERCC5. The present study reported elevated BC risk with a positive family history, showing similar results to previously reported literature (13, 14). The present study also reported that increased BC risk was linked to late menopause and early menarche, which is concordant with the literature (15). To maintain genome integrity, regulation of the NER pathway is essential, and ERCC5 is a multifunctional gene that encodes structure-specific endonucleases (16). Studies have found an association between ERCC5 genetic variations and different cancers (8). The current study reported a significant association between variant types of rs751402 and rs2094258 with an elevated risk of breast cancer. At present, very few studies are available with respect to the mentioned polymorphisms and breast cancer. Significant correlation of variant genotypes of rs751402, rs2094258, and rs873601 has been stated with colorectal cancer susceptibility (17). Pongsavee and Wisuwan (4) also reported a significant association of rs751402 with breast cancer in a Thai population. Wang et al. (7) had described no association of rs17655 with BC among the Han population of northwest China and a significant association of rs751402 with breast carcinogenesis. A meta-analysis showed that rs873601 was significantly associated with overall risk, and another meta-analysis showed that this polymorphism is involved in the development and severity of colorectal cancer (8, 18). Guo et al. (12) investigated the role of rs17655 and rs751402 in the development of gastric cancer in a Chinese population and found that the mutant genotype of rs751402 significantly increased gastric cancer risk compared to the wild type, but rs17655 did not. Several meta-analyses have found that the rs17655 polymorphism might not confer susceptibility to breast cancer, and the results are still inconsistent (6, 19). Our study showed short survival due to delayed medical aid, a diverse medical history, and an advanced disease stage. Patients with early medical aid have higher 5- year survival rates than those with delayed presentation (88% and 12%, respectively) (20). Most of the patients in the present study had advanced stages of disease because they were from rural areas and mostly lacked disease knowledge and had financial constraints to go for therapeutic options and a good diet. Looking for medical aid early, before the advanced stage, implies a better prognosis and ultimately improves survival rates.

## Conclusion

In conclusion, two SNPs (rs751402 and 2094258) may play a role in the etiology of breast cancer in Pakistan. This is the first report of the association between ERCC5 (rs751402, rs2094258, rs17655, and rs873601) and breast cancer risk in Pakistan. The literature is limited in this area; therefore, for more pronounced results, studies with larger sample sizes are needed. Furthermore, late menopause, positive ER/PR status, and a positive family history are contributing factors to breast cancer development.

## Data availability statement

The original contributions presented in the study are included in the article/**Supplementary Material**. Further inquiries can be directed to the corresponding author.

## Ethics statement

The studies involving human participants were reviewed and approved by the Research Ethics Committee of Fatima Jinnah Women University, Rawalpindi, Pakistan. The patients/participants provided their written informed consent to participate in this study.

## Author contributions

IK was involved in the execution of research and experimental work. NM was involved in concept, design, write up. AY was involved in final draft review, experimental lab, and chemicals provision. All authors listed have made a substantial, direct, and intellectual contribution to the work and approved it for publication.
