# Supplementary material for: Correlation of ERCC5 polymorphisms and linkage disequilibrium associated with overall survival and clinical outcome to chemotherapy in breast cancer
Source: Front Oncol. 2023 Jan 4;12:1091514. doi: 10.3389/fonc.2022.1091514 (PMC9846539; doi:10.3389/fonc.2022.1091514)
Supplement: Supplementary file 1 [file DataSheet_1.docx]

**Correlation of ERCC5 Polymorphisms and linkage disequilibrium associated with overall survival and clinical outcome to chemotherapy in breast cancer**

Iqra, Nosheen Masood*, Azra Yasmin

Microbiology and Biotechnology Research Lab, Department of Biotechnology, Fatima Jinnah Women University, Rawalpindi, Pakistan

*Correspondence: [dr.nosheen@fjwu.edu.pk](mailto:dr.nosheen@fjwu.edu.pk)

**Supplementary Materials**

**Table S1: PCR Primers sequence details of single nucleotide polymorphisms of ERCC5**

| **Polymorphism** | **Primers** | **Annealing Temp** | **PCR cycles** | **Reference** |
| --- | --- | --- | --- | --- |
| **rs751402** | 5'-GAGCGGGCCCATTTTCC-3'  5'TCACCGCCTCCCGGAAGAAAGA3' | 56 °C | 35 | Guo et al., 2016 |
| **rs17655** | 5'CATCTGATGGATCTTCAAGTCTA3'  5'-TCACGAGGACCATCTTCT-3' | 58 °C | 40 | Guo et al., 2016 |
| **rs2094258** | 5'CGGTGGCTCATGTCTGTA3'  5'GTGCCTCACTATGGTGTTTA3' | 63 °C | 30 | He et al., 2013 |
| **rs873601** | 5'-CTGGTATGAGCCCATCTA-3'  5'-AGTGACAAGCCTGTAGCC-3' | 62 °C | 35 | Zou & Zhao, 2015 |

**Table S2: ERCC5 germline variants and overall survival of BC patients with various survival functions**

| **SNP** | **OS** | | | |
| --- | --- | --- | --- | --- |
| **Genotypes** | **Events (n=182)** | **Median (months)** | **Log rank (P value)** | **Confidence Interval** |
| **rs17655** | | | | |
| Wild type | 17 | 25 | 0.3 | 20.5-29.4 |
| Variants | 114 | 25 |  | 20.3-29.6 |
| **Survival Functions** | | | | |
| **Positive family history** | | | | |
| Wild type | 37 | 26 | 0.5 | 19.2-32.7 |
| Variant types | 18 | 21 |  | 15.1-26.8 |
| **Used contraceptives** | | | | |
| Wild type | 29 | 25 | 0.6 | 16.2-33.7 |
| Variant types | 6 | 18 |  | 3.5-32.4 |
| **Positive ER/PR** | | | | |
| Wild type | 103 | 26 | 0.5 | 21.5-30.4 |
| Variant types | 28 | 25 |  | 19.8-30.1 |
| **rs751402** | | | | |
| Wild type | 23 | 11 | <0.001 | 6.3-15.6 |
| Variants | 159 | 26 |  | 24.6-27.3 |
| **Survival functions** | | | | |
| **Positive family history** | | | | |
| Wild type | 6 | 7 | 0.001 | 0.00-15.4 |
| Variant type | 49 | 25 |  | 21.5-28.4 |
| **Used contraceptives** | | | | |
| Wild type | 6 | 13 | 0.001 | 9.3-16.6 |
| Variant types | 29 | 26 |  | 24.2-27.7 |
| **Positive ER/PR** | | | | |
| Wild type | 17 | 11 | 0.001 | 4.2-17.7 |
| Variant types | 114 | 26 |  | 24.5-27.4 |
| **rs2094258** | | | | |
| Wild type | 71 | 27 | P<0.001 | 25.3-28.6 |
| Variants | 111 | 21 |  | 16.4-25.5 |
| **Survival functions** | | | | |
| **Positive family history** | | | | |
| Wild type | 21 | 27 | 0.006 | 24.7-29.2 |
| Variant types | 34 | 21 |  | 15.2-26.7 |
| **Used contraceptives** | | | | |
| Wild type | 13 | 27 | 0.01 | 16.4-37.5 |
| Variant types | 22 | 18 |  | 11.4-24.5 |
| **Positive ER/PR** | | | | |
| Wild type | 50 | 27 | 0.006 | 25-28 |
| Variant types | 81 | 24 |  | 18-29 |
| **rs873601** | | | | |
| Wild type | 140 | 25 | 0.08 | 21.1-28.8 |
| Variants | 42 | 26 |  | 16.4-35.5 |
| **Survival functions** | | | | |
| **Positive family history** | | | | |
| Wild type | 48 | 25 | 0.09 | 21.1-28.8 |
| Variant types | 7 | 26 |  | 1.0-58.3 |
| **Used contraceptives** | | | | |
| Wild type | 26 | 25 | 0.1 | 17.8-32 |
| Variant types | 9 | 18 |  | 6.3-29.6 |
| **Positive ER/PR** | | | | |
| Wild type | 99 | 25 | 0.9 | 21-28.4 |
| Variant types | 33 | 27 |  | 15.9-38 |

**
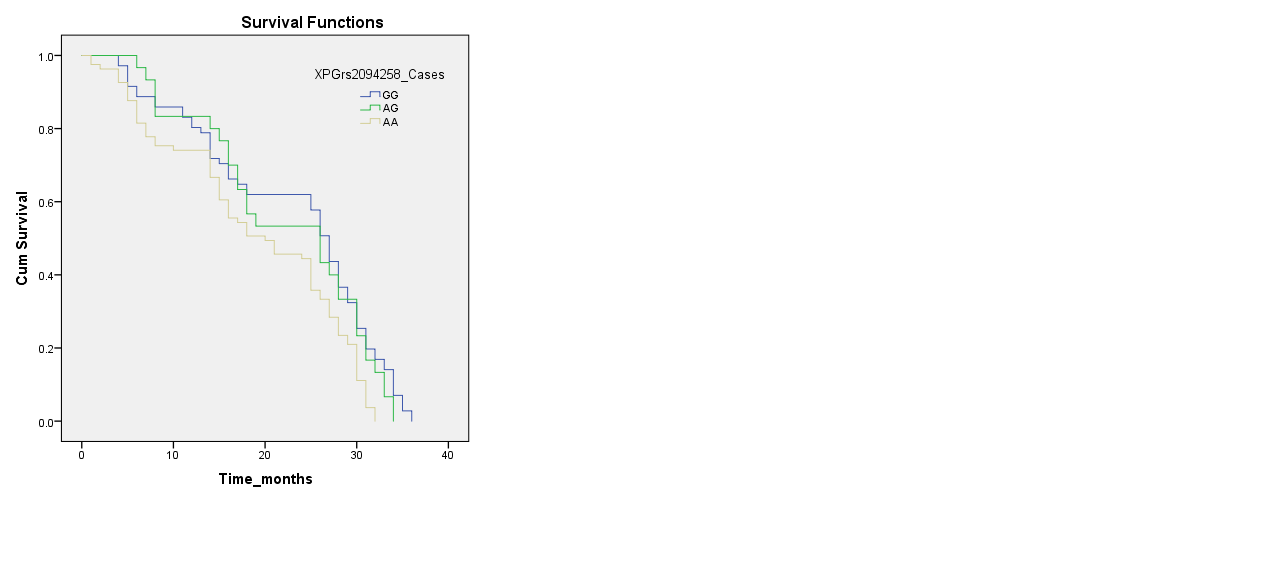
**
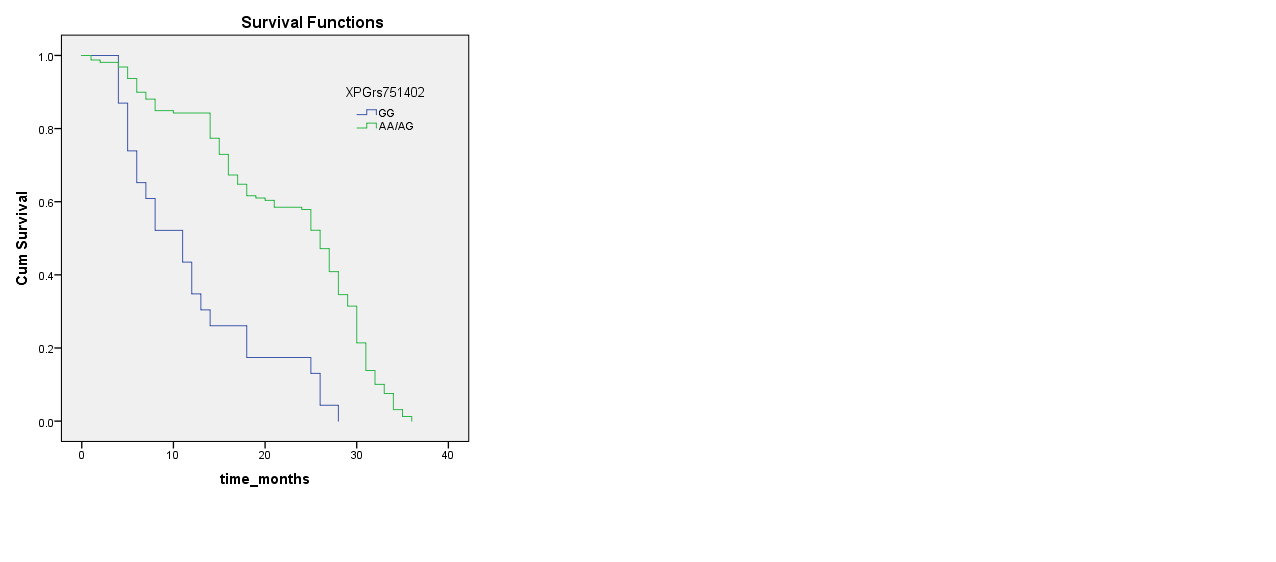


A) B)

**
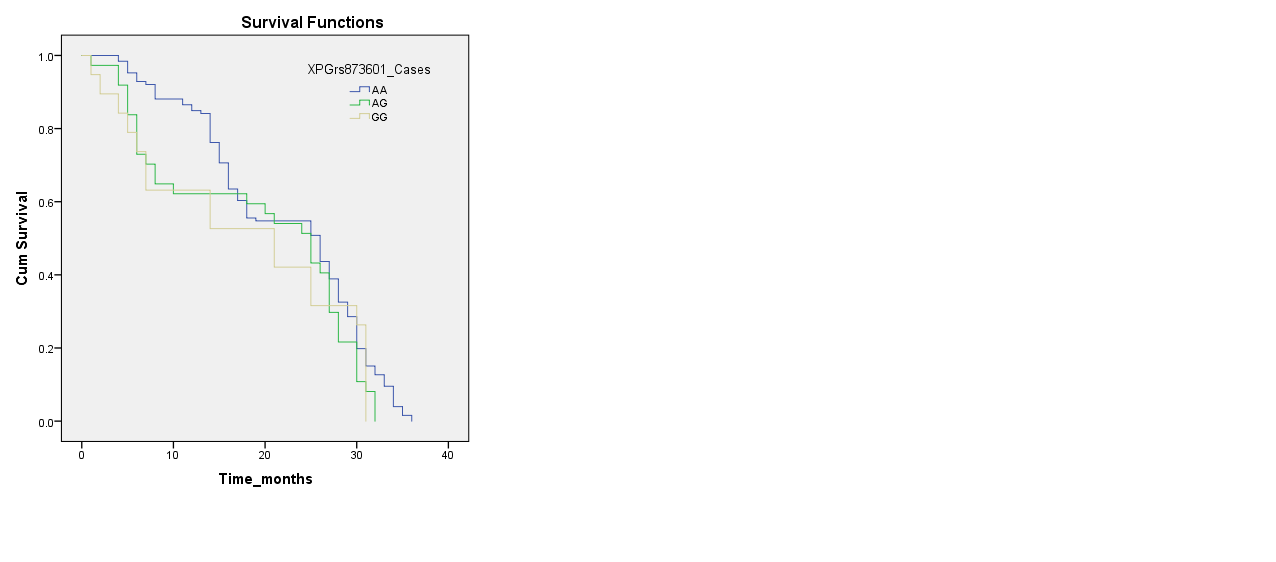
**
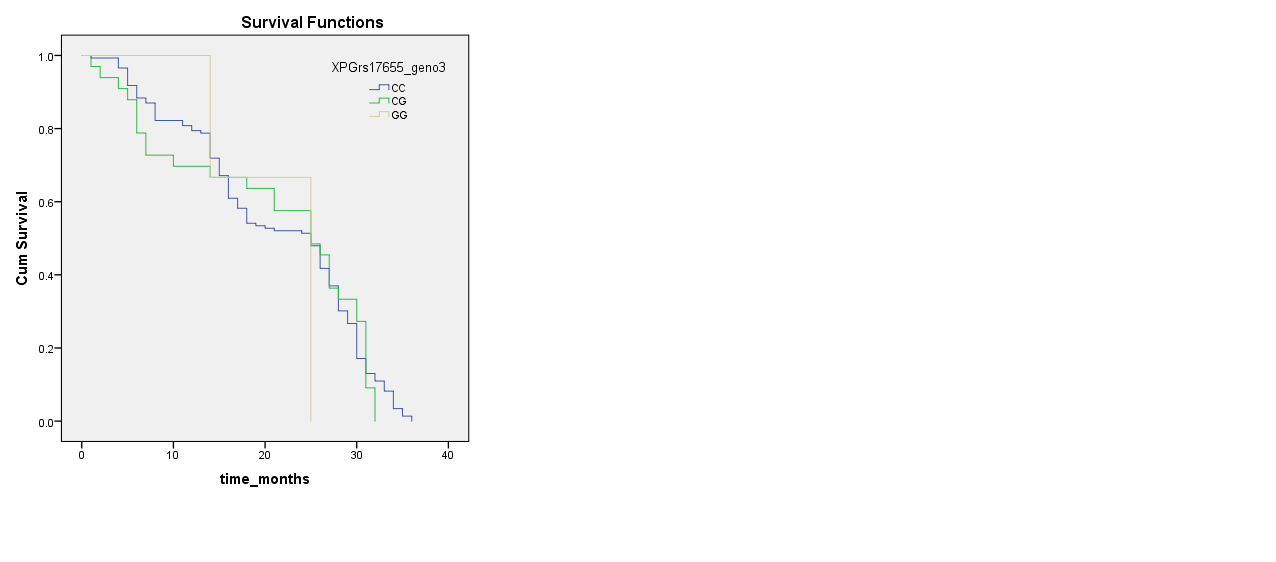


C) D)  **
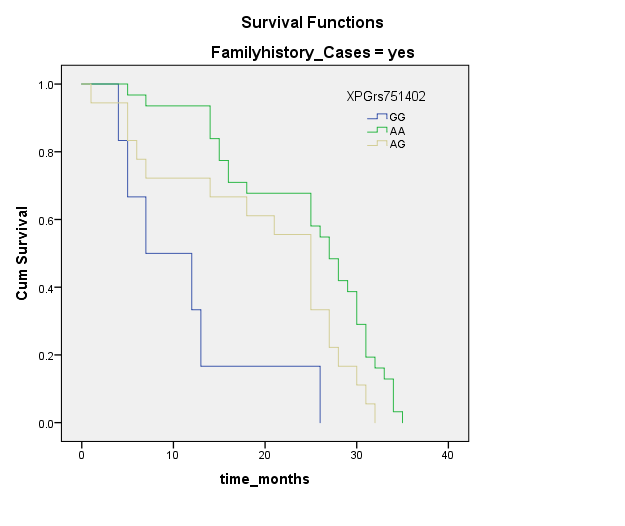
**
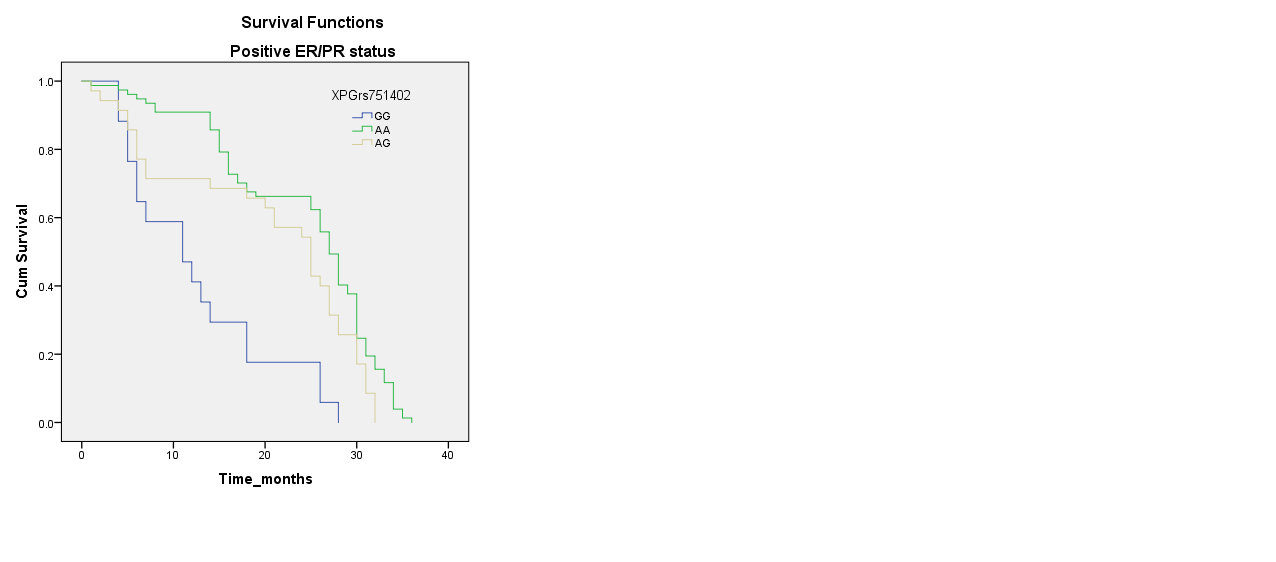


E) F)


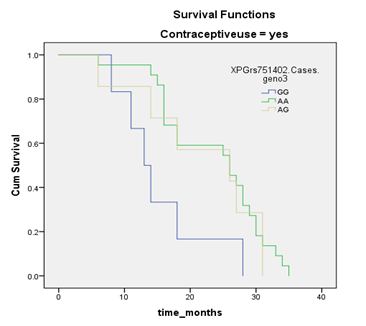

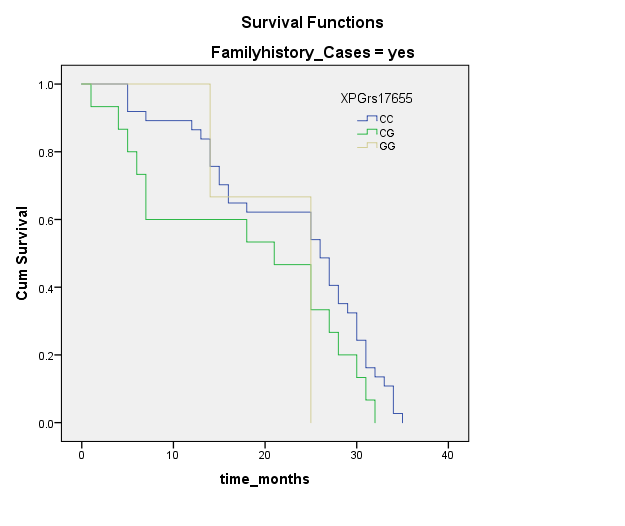


G) H)

**
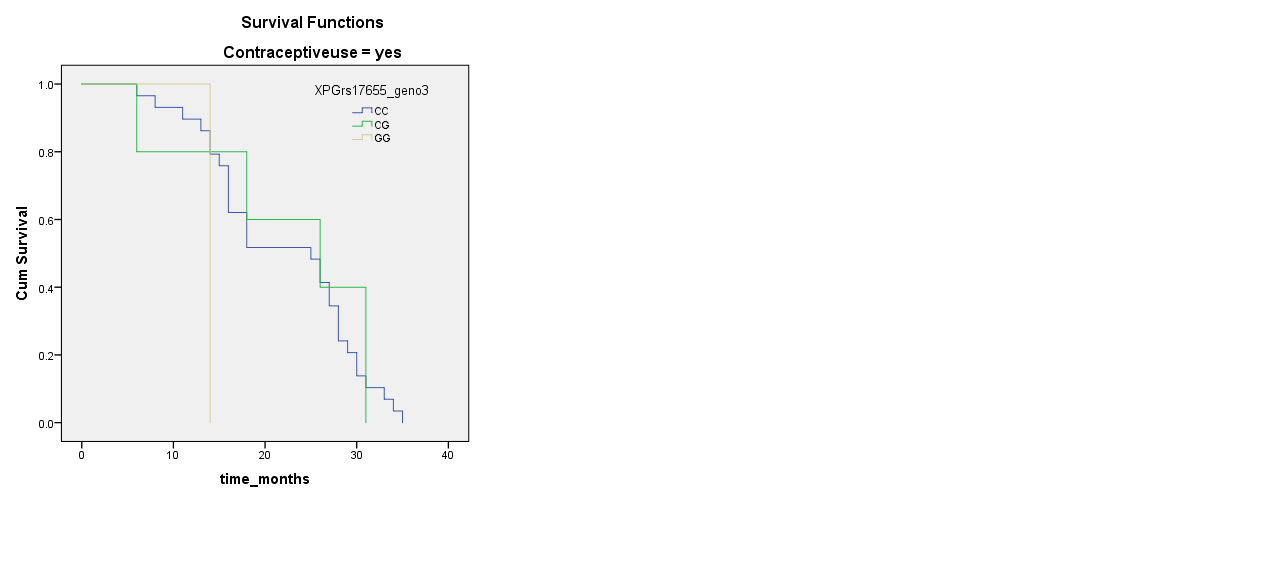
**
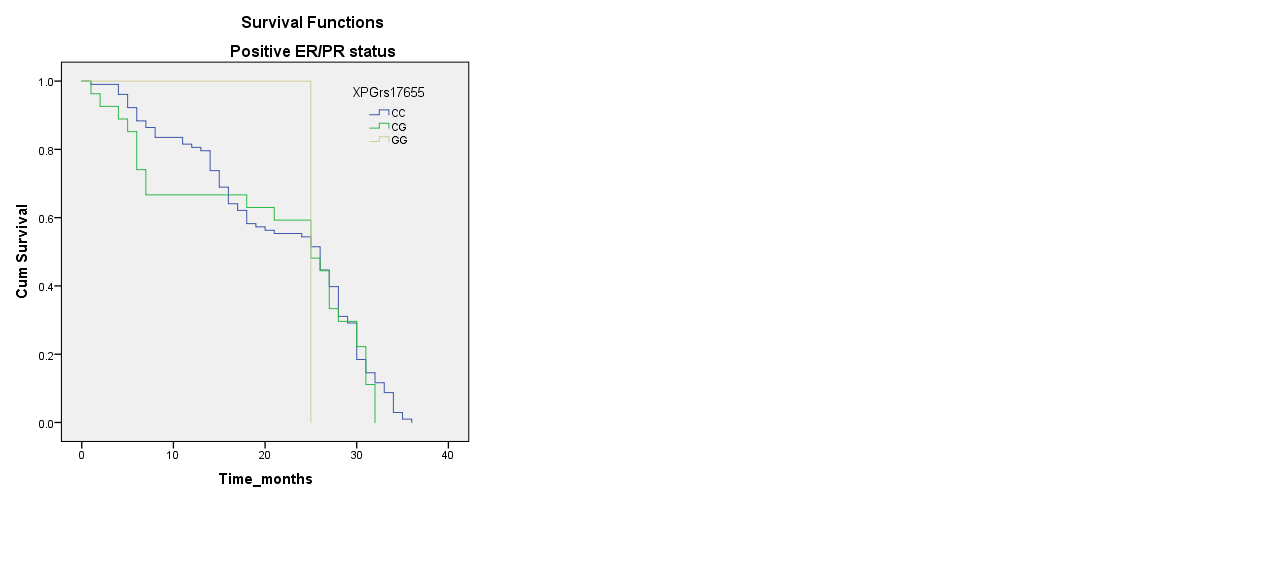


I) J)


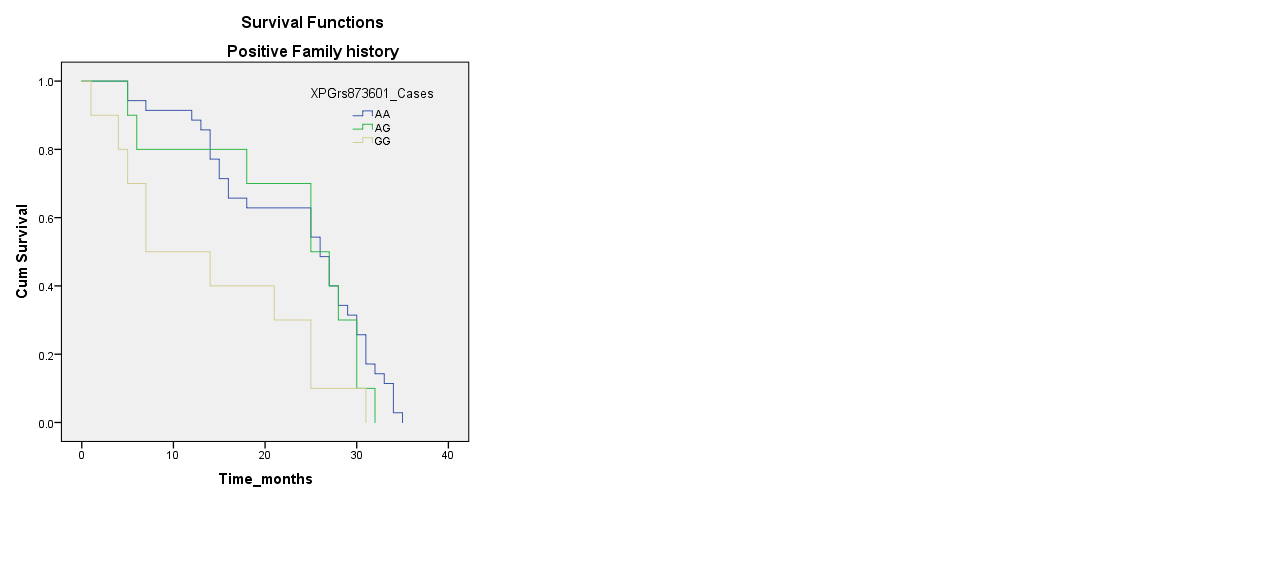

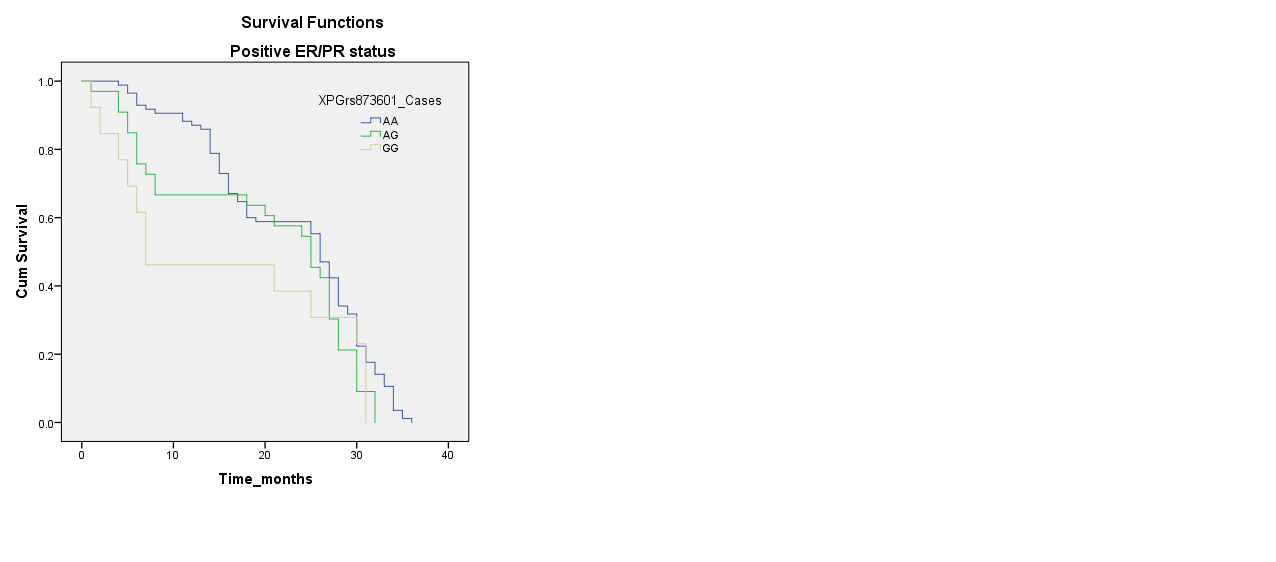


K) L)

**
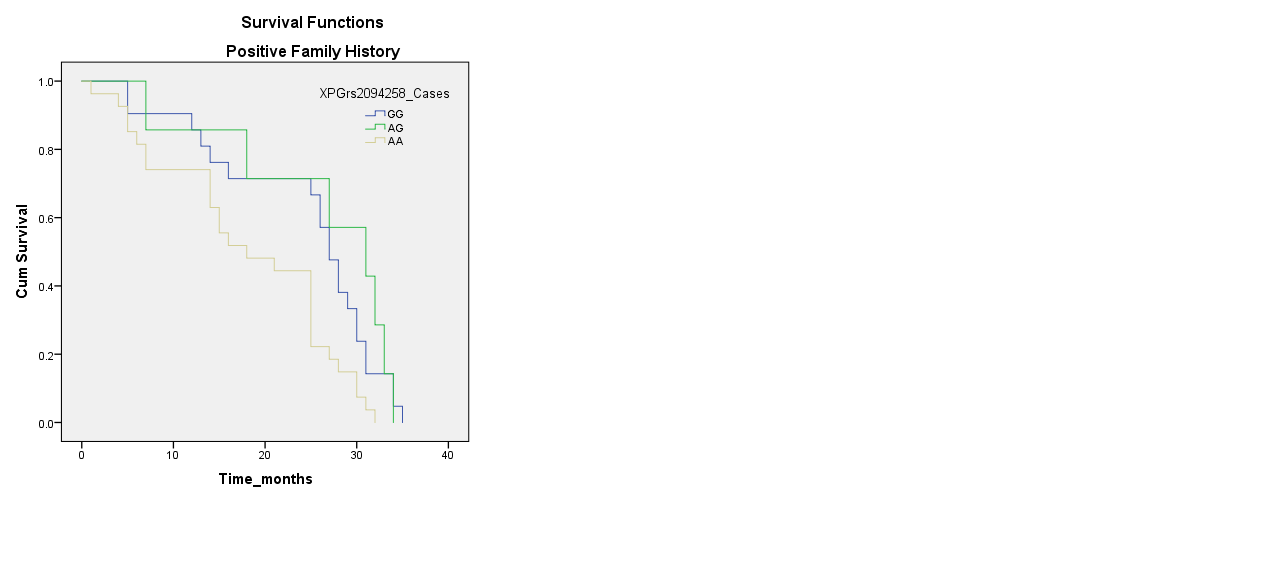
**
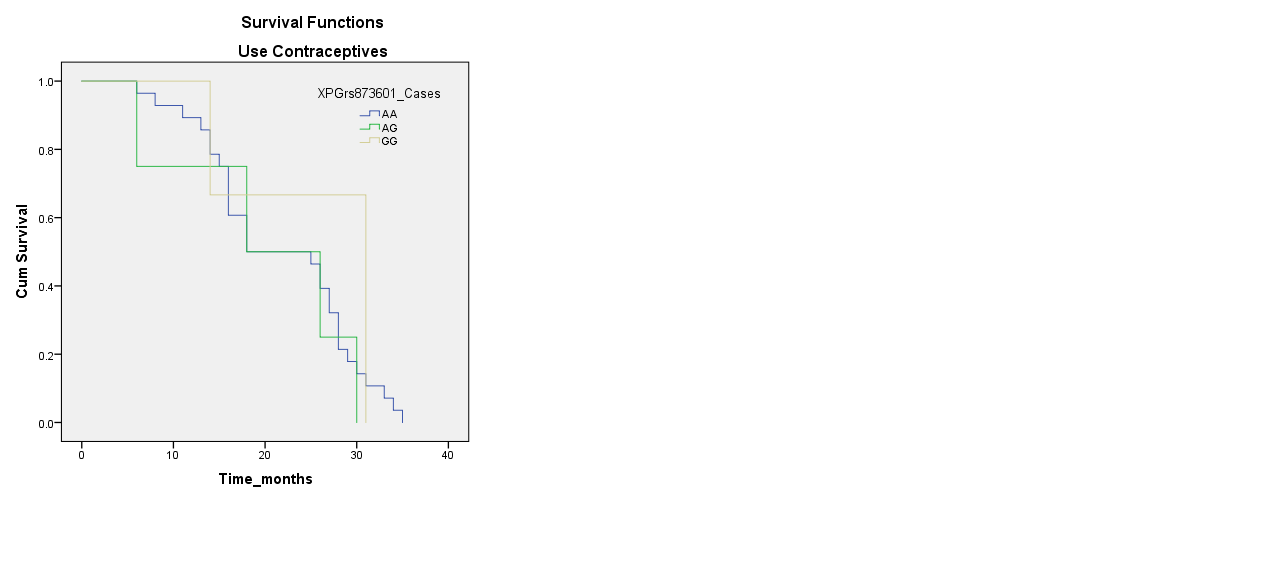


M) N)


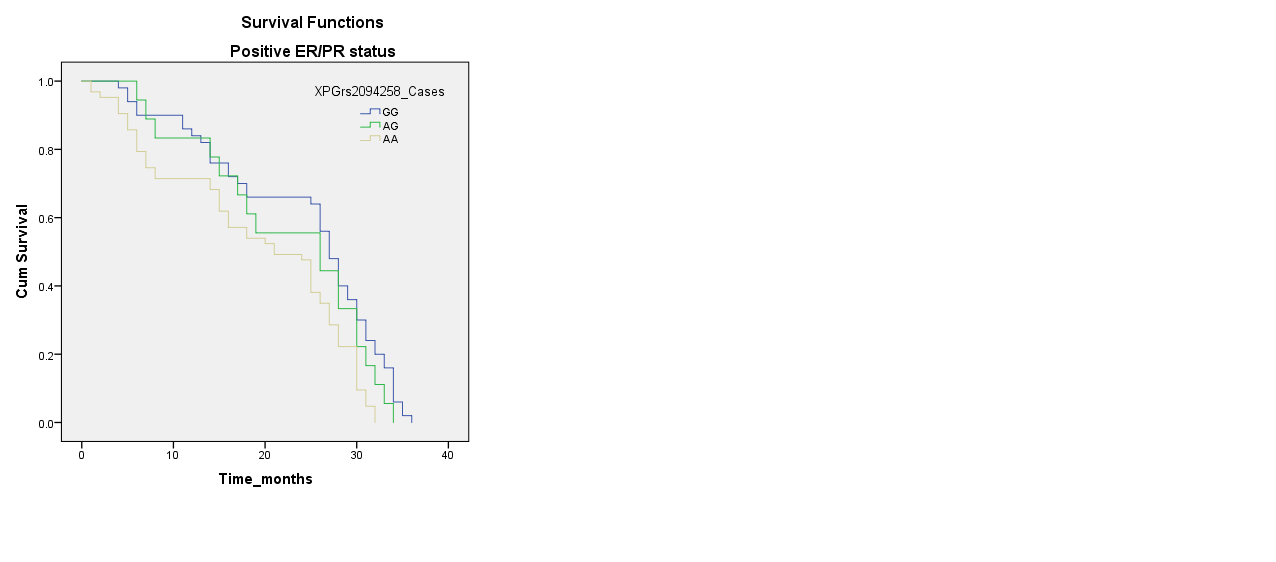

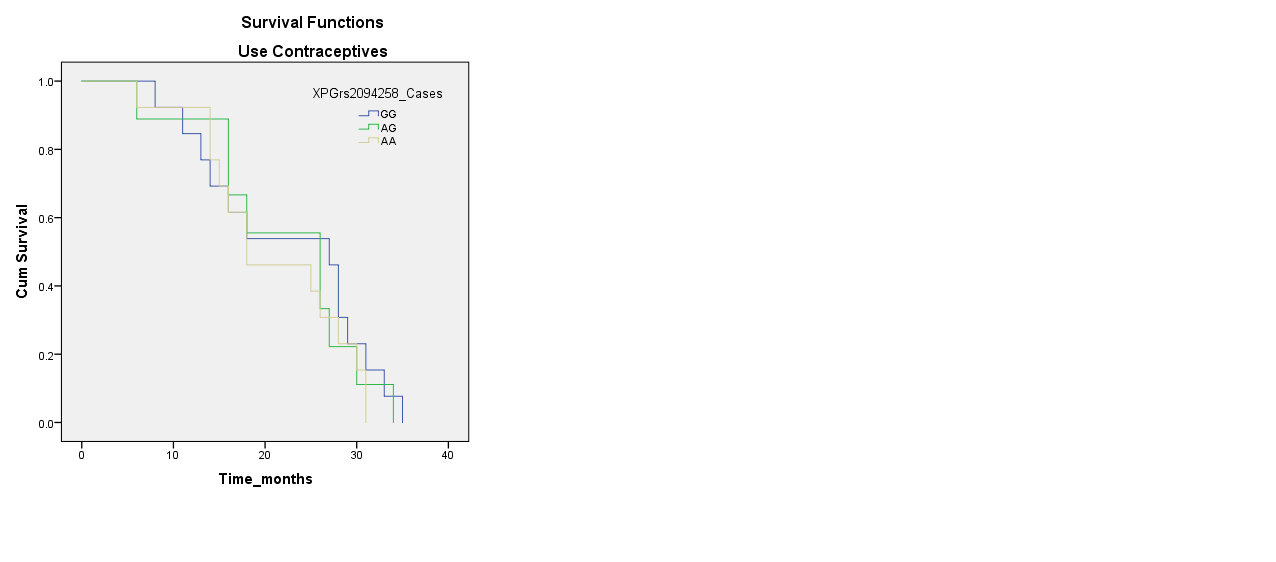


O) P)

**Figure S1:** Kaplan-Meier Curves illustarting; A-D) OS of ERCC5 rs751402, rs2094258, rs17655 and rs873601 variants with BC over period of 36 months E) OS of rs751402 variants adjusted for BC cases who had positive family history of cancer. F) OS of rs751402 variants adjusted for BC cases who had positiveER/PR. G) OS of rs751402 variants adjusted for BC cases who used contraceptives. H-J) OS of rs17655 variants adjusted for BC cases who had positive family history ER+PR status and used contraceptives. K-M) OS of rs873601 variants adjusted for BC cases who had positive family history ER+PR status and used contraceptives. N-P) OS of rs2094258 variants adjusted for BC cases who had positive family history ER+PR status and used contraceptives.
